# Supplementary material for: Spatial analysis of accessibility to healthcare-related facilities in Tokyo Metropolis using geographic information systems
Source: PLoS One. 2026 May 29;21(5):e0350130. doi: 10.1371/journal.pone.0350130 (PMC13221066; doi:10.1371/journal.pone.0350130)
Supplement: S2 File — e-stat terms of use (English). (DOCX) [file pone.0350130.s002.docx]

e-Stat Terms of Use | Portal Site of Official Statistics of Japan

[**https://www.e-stat.go.jp/terms-of-use**](https://www.e-stat.go.jp/terms-of-use)

(Translated by chat GPT 5.2)

**--**

**Portal Site of Official Statistics of Japan (e-Stat) Terms of Use**

The information published on this website (hereinafter referred to as the “Content”) may be freely used by anyone, including for reproduction, public transmission, translation, adaptation (including modification), and other uses, in accordance with items (1) through (6) below. Commercial use is also permitted.

In addition, numerical data, simple tables, graphs, and similar materials are not subject to copyright protection; therefore, these materials are not subject to these usage rules and may be used freely.

By using the Content, users are deemed to have agreed to these usage rules.

**1) Indication of Source**

**a.** When using the Content, please indicate the source. Examples of how to indicate the source are as follows:

**(Examples of source indication)**
Source: Portal Site of Official Statistics of Japan (e-Stat) (<https://www.e-stat.go.jp/>)
Source: “Results of the ○○ Survey” (Ministry A), etc.

**b.** If you use the Content after editing, processing, or otherwise modifying it, please indicate that you have made such edits or modifications in addition to the source above.
Note that you must not publish or use edited/processed information in a manner that makes it appear as though it was created by the national government (or ministries/agencies, etc.).

**(Examples of indication when editing/processing the Content)**
Created by processing “Results of the ○○ Survey” (Ministry A)
Created by ○○ Co., Ltd. based on “Results of the ○○ Survey” (Ministry A), etc.

**2) Do Not Infringe Third-Party Rights**

**a.** Some Content may be subject to copyrights or other rights held by third parties (i.e., parties other than the national government; the same applies hereinafter). For Content in which third parties hold copyrights, or Content in which third parties hold rights other than copyright (e.g., portrait rights or publicity rights for photographs), unless it is clearly indicated that rights clearance has been completed, users must obtain permission for use from the relevant third parties at their own responsibility.

**b.** Among the Content for which third parties hold rights, some may directly or indirectly indicate or suggest that third parties hold such rights through source indications, etc., while others may not clearly identify or specify which parts are subject to third-party rights. Users must confirm this at their own responsibility before use.

**c.** For Content obtained through API (Application Programming Interface) linkage with external databases and the like, please comply with the usage conditions of the respective providers.

**d.** Even where third parties hold copyrights or other rights, there may be cases where use is permitted without obtaining permission under the Copyright Act, such as quotation/citation that is legally allowed.

**3) Content to Which These Usage Rules Do Not Apply**

These usage rules do not apply to the following Content:

**a.** Symbol marks, logos, and character designs representing organizations or specific projects/programs.

**b.** Content for which it is clearly stated (together with a specific and reasonable explanation of grounds) that different usage rules apply.

(Content for which different usage rules apply is listed in the appendix to these usage rules.)

**4) Governing Law and Jurisdiction**

**a.** These usage rules shall be interpreted in accordance with the laws of Japan.

**b.** With respect to the use of Content under these usage rules and any disputes relating to these usage rules, the district court having jurisdiction over the location of the organization that publishes the Content or these usage rules relevant to the dispute shall be the exclusive court of first instance agreed upon by the parties.

**5) Disclaimer**

**a.** The national government shall not be liable for any actions taken by users using the Content (including the use of information created by editing/processing the Content).

**b.** The Content may be changed, moved, deleted, etc., without prior notice.

**6) Miscellaneous**

**a.** These usage rules do not restrict uses permitted under the Copyright Act, such as legally allowed quotation/citation.

**b.** These usage rules were established on January 29, 2016. They conform to the Government of Japan Standard Terms of Use (Version 2.0). These usage rules may be amended in the future. If you have already been using the Content in accordance with a previous version of the Government of Japan Standard Terms of Use, the conditions of that prior version will continue to apply.

**c.** These usage rules are compatible with the Creative Commons Attribution 4.0 International license (“CC BY”) as set forth at:
<https://creativecommons.org/licenses/by/4.0/legalcode.ja>
Content to which these usage rules apply may also be used in accordance with CC BY.

**Appendix**

Different usage rules apply. For details, please refer to the linked pages.

- Use of the logo of the Portal Site of Official Statistics of Japan (e-Stat)
- Use of the logos of the Microdata Utilization Portal Site for Official Statistics (miripo) and the Electronic Application Portal for Microdata Use (e-Micro)
- Use of the API function
- Use of the Statistical GIS (Statistical Geographic Information System)
- Use of the Electronic Application Portal for Microdata Use (e-Micro)
- Use of the Questionnaire Information Provision Function
