# Supplementary material for: Spatial analysis of accessibility to healthcare-related facilities in Tokyo Metropolis using geographic information systems
Source: PLoS One. 2026 May 29;21(5):e0350130. doi: 10.1371/journal.pone.0350130 (PMC13221066; doi:10.1371/journal.pone.0350130)
Supplement: S4 File — National land numerical information download site terms of use (English). (DOTM) [file pone.0350130.s004.dotm]

Terms of Use for the National Land Numerical Information Download Site

(Translated by ChatGPT 5.2)

# Terms of Use for Content on the National Land Numerical Information Download Site

*(Government of Japan Standard Terms of Use–Compliant Version)*
*(Established: April 1, 2020)*

**1. Use of Content on the National Land Numerical Information Download Site**

The information published on this National Land Numerical Information Download Site (hereinafter, the “Content”) may be freely used by anyone, in accordance with items (1) through (6) below, including reproduction, public transmission, translation, and adaptation such as modification and transformation. Commercial use is also permitted. In addition, simple tables, graphs, and the like are not subject to copyright protection; therefore, these items are not subject to these rules and may be used freely.

Users of the Content are deemed to have agreed to these rules.

**(1) Indication of Source**

**a.** When using the Content, please indicate the source. Examples are as follows:

**(Examples of source indication)**
Source: Ministry of Land, Infrastructure, Transport and Tourism (MLIT), National Land Numerical Information Download Site (URL of the relevant page)
Source: “National Land Numerical Information (○○ data)” (MLIT) (URL of the relevant page) (Retrieved on: ○○/○○/○○)

**b.** If you use the Content after editing or processing it, please indicate that you have edited/processed it in addition to the source above.
You must not publish or use edited/processed information in a manner that makes it appear as though it was created by the national government (or ministries/agencies, etc.).

**(Examples when editing/processing the Content)**
Created by processing “National Land Numerical Information (○○ data)” (MLIT) (URL of the relevant page)
Created by ○○ Co., Ltd. based on “National Land Numerical Information (○○ data)” (MLIT) (URL of the relevant page)

**(2) Do Not Infringe Third-Party Rights**

**a.** Some Content may be subject to copyrights or other rights held by third parties (i.e., parties other than the national government; the same applies hereinafter). For Content in which third parties hold copyrights, or Content in which third parties hold rights other than copyright (e.g., portrait rights or publicity rights in photographs), unless it is clearly indicated that rights clearance has been completed, users must obtain permission from the relevant third parties at their own responsibility.

**b.** For Content in which third parties hold rights, some materials may directly or indirectly indicate or suggest such third-party rights via source indications, etc., while others may not clearly identify the portions subject to third-party rights. Users must confirm this at their own responsibility before use.

**c.** Even for Content in which third parties hold copyrights or other rights, there may be cases where use is permitted without permission under the Copyright Act, such as legally permitted quotation/citation.

**(3) Content to Which These Rules Do Not Apply**

These rules do not apply to the following Content:

**a.** Symbol marks, logos, and character designs representing organizations or specific projects/programs.

**b.** Content for which it is clearly stated (together with a specific and reasonable explanation of grounds) that different usage rules apply.
(Content for which different rules apply is listed in the appendix to these rules.)

**(4) Governing Law and Jurisdiction**

**a.** These rules shall be interpreted in accordance with the laws of Japan.

**b.** With respect to use of the Content under these rules and any disputes relating to these rules, the district court having jurisdiction over the location of the organization that publishes the Content or these rules relevant to the dispute shall be the exclusive court of first instance agreed upon by the parties.

**(5) Disclaimer**

**a.** The national government shall not be liable for any actions taken by users using the Content (including the use of information created by editing/processing the Content).

**b.** The Content may be changed, moved, deleted, etc., without prior notice.

**(6) Miscellaneous**

**a.** These rules do not restrict uses permitted under the Copyright Act, such as legally permitted quotation/citation.

**b.** These rules were established on April 1, 2020. They conform to the Government of Japan Standard Terms of Use (Version 2.0). These rules may be amended in the future. If you have already been using the Content in accordance with a previous version of the Government of Japan Standard Terms of Use, the conditions of that prior version will continue to apply.

**c.** These rules are compatible with the Creative Commons Attribution 4.0 International license (“CC BY”). Content to which these rules apply may also be used under CC BY.

https://creativecommons.org/licenses/by/4.0/legalcode.ja

**Content Subject to Different Rules**

Depending on the Content, the usage conditions permitted by the original data provider may differ from these rules. Therefore, if the “Usage Restrictions” field on an individual National Land Numerical Information page states that it is governed by terms of use other than these rules, you must comply with those stated terms.

**Content in Which Third Parties Hold Copyrights or Other Rights**

Data that includes the following statement in the “License Conditions for Use of This Data” field on an individual National Land Numerical Information page is published with permission from the original data provider to make the data available under these rules:

**<Applicable Rules>**
Terms of Use for Content on the National Land Numerical Information Download Site (Government Standard Terms–Compliant Version)

**<Conditions of Use>**
In accordance with the applicable terms of use (Open Data)

**Terms of Use for Content on the National Land Numerical Information Download Service**

*(Version Conforming to the Former National Land Information Terms of Use)*
*(Established: April 1, 2020; Revised: July 1, 2020)*

**Article 1 (Handling of Copyrights, etc.)**

“National Land Numerical Information” is a collection of databases of fundamental spatial information related to the national land. In principle, each dataset is a derivative work created by the Real Estate and Construction Economy Bureau of the Ministry of Land, Infrastructure, Transport and Tourism (MLIT) by extracting elements from original works/intellectual property that have original copyright holders and other rights holders (generally other than the MLIT bureau) and by assigning location data, within the scope permitted by those rights holders.

Accordingly, each indicator/dataset has original authors and various rights holders, and the permitted scope of use differs by dataset. Please comply with the “License Conditions for Use of This Data” provided for each dataset and all relevant laws and regulations in Japan, and do not infringe the rights of the original copyright holders. Publication may be suspended or terminated without prior notice at the request of original authors or other rights holders.

Based on the government’s open-data policy, the MLIT bureau aims to expand datasets that allow commercial use. Each group of indicators in “National Land Numerical Information” displays one of the following two licenses:

**(a) “Commercial Use Permitted”**: Use for commercial purposes (including redistribution of copies) is permitted within the scope of permission from the original authors/rights holders, provided that the source, processor/editor, etc. are indicated.

**(b) “Non-Commercial”**: Use is permitted only for non-commercial purposes within the scope of permission from the original authors/rights holders, provided that the source, processor/editor, etc. are indicated (however, redistribution of copies is excluded).

If, when using the data for official purposes by administrative bodies, etc., the permissible scope is unclear, please contact the Secretariat of the National Land Information Provision Site.

In addition, all users of “National Land Numerical Information” and its processed outputs are requested to communicate and pass on, for each dataset, the dataset’s license and the contents of these terms, including the incompleteness of the data, the limits of applicability, the location of rights, and the handling of copyrights, etc.

**Article 2 (Limits of Applicability of “National Land Numerical Information” and Their Communication/Inheritance)**

1. “National Land Numerical Information” was created by acquiring coordinates faithfully based on each source material, using 1:25,000 topographic maps published by the Geospatial Information Authority of Japan (GSI) or their digital products as base maps. Therefore, it contains spatial and temporal errors originating from those sources. Spatially, where the source is address information, representative points may have tolerances at the block/aza (neighborhood) level (roughly several hundred meters). Temporally, there may be a time lag of approximately 1–5 years. Accordingly, all users, from intermediate processors to end users, must understand the applicability limits of each indicator and independently determine whether it suits their purposes, and must also communicate/pass on these limits to prevent misuse.
2. “National Land Numerical Information” is created based on source materials that reached the secretariat at the time of compilation. Due to non-receipt of source materials, some parts of the data may be missing or outdated even if the product specifications state otherwise. Users should use the data after cross-checking with the original sources, etc.
3. “National Land Numerical Information” consists of data created primarily for national land planning and land/real-estate-related operations and is made publicly available secondarily. Therefore, it is not intended for uses requiring high precision such as navigation, public surveying, and various certifications. Moreover, even aside from temporal/spatial precision, it may not be suitable for the user’s intended purpose. Users must, at their own responsibility, carefully examine suitability before use.
4. Not only compilers but also end users of deliverables/services must ensure that the above applicability limits are communicated and passed on, for example by stating them as assumptions/conditions prior to use.

**Article 3 (Indication of Source)**

1. When using “National Land Numerical Information,” please indicate the source. Examples are as follows:

**(Examples of source indication)**
Source: MLIT National Land Numerical Information Download Site (URL of the relevant page)
Source: National Land Numerical Information (○○ data) (MLIT) (URL of the relevant page) (Retrieved on: ○○/○○/○○)

If you use “National Land Numerical Information” after editing/processing, please indicate that you have edited/processed it in addition to the source above. You must not publish or use edited/processed information in a manner that makes it appear as though it was created by the national government (or the original authors).

**(Examples when editing/processing the data)**
Created by processing “National Land Numerical Information (○○ data)” (MLIT) (URL of the relevant page)
Created by ○○ Co., Ltd. based on “National Land Numerical Information (○○ data)” (MLIT) (URL of the relevant page)

1. Even for “Non-Commercial” National Land Numerical Information, results of spatial calculations performed by GIS (that are not databases), etc., may be used by clearly indicating the source and the name of the processor/editor. However, if the data to be used is a database in GIS format or Excel format, etc., there is a risk of infringing database copyright held by the original authors/rights holders. Please contact the National Land Information Download Service Secretariat.

**Article 4 (Use of “National Land Numerical Information”)**

1. “National Land Numerical Information” may be used free of charge. However, communication charges and other costs necessary to use it shall be borne by the user.
2. Users of “National Land Numerical Information” are deemed to have agreed to these terms.
3. Any use of “National Land Numerical Information” for purposes or by means/methods that violate any laws, cabinet orders, or any other laws/regulations/ordinances is strictly prohibited. Use for purposes or by means/methods that infringe the rights of others, or that violate public order and morals, is also strictly prohibited.
4. Decisions regarding the purpose and method of use are solely the responsibility of the user, and the MLIT shall not be involved in any way.
5. For some datasets, restrictions may apply to the purpose and/or method of use due to the need to protect copyrights in the source data. Please be sure to follow the explanations for each dataset.

**Article 5 (Disclaimer)**

Regardless of the cause, the user shall bear all responsibility for any damages suffered by the user or any third party arising from use of “National Land Numerical Information,” and neither the MLIT nor the providers of the source materials shall bear any liability.

**Article 6 (Miscellaneous)**

1. The MLIT may change or delete the contents of “National Land Numerical Information,” or suspend, pause, or discontinue its provision, without prior notice.
2. Use of “National Land Numerical Information” under these terms shall be governed by Japanese law and these terms.
3. The court of jurisdiction for disputes relating to these terms shall be the Tokyo District Court.
4. While maximum efforts are made to verify data quality, errors may still be included. If you find errors, please contact the inquiry desk with details.
5. These terms may be changed without prior notice.

**Terms of Use for Content on the Location Reference Information Download Service**

*(Established: April 1, 2020)*

**1. Use of Content on the Location Reference Information Download Service**

The information published on this Location Reference Information Download Service (hereinafter, the “Content”) may be freely used by anyone, in accordance with items (1) through (6) below, including reproduction, public transmission, translation, and adaptation such as modification and transformation. Commercial use is also permitted. In addition, simple tables, graphs, and the like are not subject to copyright protection; therefore, these items are not subject to these rules and may be used freely.

Users of the Content are deemed to have agreed to these rules.

**(1) Indication of Source**

**a.** When using the Content, please indicate the source. Examples are as follows:

**(Examples of source indication)**
Source: “Location Reference Information Download Service” (MLIT) (URL of the relevant page)

**b.** If you use the Content after editing/processing it, please indicate that you have edited/processed it in addition to the source above. You must not publish or use edited/processed information in a manner that makes it appear as though it was created by the national government (or ministries/agencies, etc.).

**(Examples when editing/processing the Content)**
Created by processing “Location Reference Information Download Service” (MLIT) (URL of the relevant page)
Created by ○○ Co., Ltd. based on “Location Reference Information Download Service” (MLIT) (URL of the relevant page)

**(2) Do Not Infringe Third-Party Rights**

(Identical in substance to the “Do Not Infringe Third-Party Rights” section above.)

**(3) Content to Which These Rules Do Not Apply**

These rules do not apply to symbol marks, logos, and character designs representing organizations or specific projects/programs.

**(4) Governing Law and Jurisdiction**

(Identical in substance to the “Governing Law and Jurisdiction” section above.)

**(5) Disclaimer**

(Identical in substance to the “Disclaimer” section above.)

**(6) Miscellaneous**

**a.** These rules do not restrict uses permitted under the Copyright Act, such as legally permitted quotation/citation.
**b.** These rules were established on April 1, 2020. They conform to the Government of Japan Standard Terms of Use (Version 2.0). They may be amended in the future, and prior-version conditions continue to apply for existing uses.
**c.** These rules are compatible with CC BY, and Content subject to these rules may also be used under CC BY.

https://creativecommons.org/licenses/by/4.0/legalcode.ja

**Terms of Use for Content of the National Land Survey**

*(Land Classification Survey / Water Survey)*
*(Established: April 1, 2020)*

**1. Use of Content of the National Land Survey (Land Classification Survey / Water Survey)**

The information published for the National Land Survey (Land Classification Survey / Water Survey) (hereinafter, the “Content”) may be freely used by anyone, in accordance with items (1) through (7) below, including reproduction, public transmission, translation, and adaptation such as modification and transformation. Commercial use is also permitted. In addition, simple tables, graphs, and the like are not subject to copyright protection; therefore, these items are not subject to these rules and may be used freely.

Users of the Content are deemed to have agreed to these rules.

**(1) Indication of Source**

**a.** When using the Content, please indicate the source. Examples are as follows:

**(Examples of source indication)**
Source: MLIT National Land Survey (Land Classification Survey / Water Survey) (URL of the relevant page)
Source: 1:50,000 Land Classification Basic Survey (Geomorphological Classification Map) “Northwest Tokyo” Tokyo Metropolis (1998) (MLIT) (URL of the relevant page)

**b.** If you use the Content after editing/processing it, please indicate that you have edited/processed it in addition to the source above. You must not publish or use edited/processed information in a manner that makes it appear as though it was created by the national government (or ministries/agencies, etc.).

**(Examples when editing/processing the Content)**
Created by processing “1:50,000 Land Classification Basic Survey (Geomorphological Classification Map) ‘Northwest Tokyo’ Tokyo Metropolis (1998)” (MLIT) (URL of the relevant page)
Created by ○○ Co., Ltd. based on “1:50,000 Land Classification Basic Survey (Geomorphological Classification Map) ‘Northwest Tokyo’ Tokyo Metropolis (1998)” (MLIT) (URL of the relevant page)

**(2) Do Not Infringe Third-Party Rights**

(Identical in substance to the “Do Not Infringe Third-Party Rights” section above.)

**(3) Content Subject to Restrictions Under Individual Laws**

**a.** Some Content may be subject to restrictions under individual laws. Please pay particular attention to the laws described below. For details, please refer to the linked pages.

- The results of the following surveys use GSI’s basic survey results as background maps:
  the 1:200,000 Land Classification Basic Survey and Land Conservation Basic Survey; the 1:200,000 Seamless Land Conservation Map; the 1:50,000 Prefectural Land Classification Basic Survey; Land History Survey; Disaster-Type Land Conservation Survey; Major Water System Survey; Prefectural Water Survey (Current Water Use Map); Groundwater Survey (Groundwater Map); etc.
  Therefore, for reproduction/use of these survey results including the background map under the Survey Act, approval from the Geospatial Information Authority of Japan is required.

**(4) Content to Which These Rules Do Not Apply**

These rules do not apply to symbol marks, logos, and character designs representing organizations or specific projects/programs.

**(5) Governing Law and Jurisdiction**

(Identical in substance to the “Governing Law and Jurisdiction” section above.)

**(6) Disclaimer**

(Identical in substance to the “Disclaimer” section above.)

**(7) Miscellaneous**

**a.** These rules do not restrict uses permitted under the Copyright Act, such as legally permitted quotation/citation.
**b.** These rules were established on April 1, 2020, conform to the Government of Japan Standard Terms of Use (Version 2.0), and may be amended in the future; prior-version conditions continue to apply for existing uses.
**c.** These rules are compatible with CC BY, and Content subject to these rules may also be used under CC BY.

https://creativecommons.org/licenses/by/4.0/legalcode.ja

**Terms of Use for Content of the National Land Information Web Mapping System**

*(Established: April 1, 2020)*

**1. Use of Content of the National Land Information Web Mapping System**

The information published on this National Land Information Web Mapping System (hereinafter, the “Content”) may be freely used by anyone, in accordance with items (1) through (6) below, including reproduction, public transmission, translation, and adaptation such as modification and transformation. Commercial use is also permitted. In addition, simple tables, graphs, and the like are not subject to copyright protection; therefore, these items are not subject to these rules and may be used freely.

Users of the Content are deemed to have agreed to these rules.

**(1) Indication of Source**

**a.** When using the Content, please indicate the source. Example:

Source: National Land Information Web Mapping System (MLIT) (URL of the relevant page)

**b.** If you use the Content after editing/processing it, please indicate that you have edited/processed it in addition to the source above. You must not publish or use edited/processed information in a manner that makes it appear as though it was created by the national government (or ministries/agencies, etc.).

**(Examples when editing/processing the Content)**
Created by processing National Land Information Web Mapping System (MLIT) (URL of the relevant page)
Created by ○○ Co., Ltd. based on National Land Information Web Mapping System (MLIT) (URL of the relevant page)

**(2) Do Not Infringe Third-Party Rights**

**a.** Some Content may be subject to copyrights or other rights held by third parties (i.e., parties other than the national government; the same applies hereinafter). For Content in which third parties hold copyrights, or Content in which third parties hold rights other than copyright (e.g., portrait rights or publicity rights in photographs), unless it is clearly indicated that rights clearance has been completed, users must obtain permission from the relevant third parties at their own responsibility.

**b.** For Content in which third parties hold rights, some materials may directly or indirectly indicate or suggest such third-party rights via source indications, etc., while others may not clearly identify the portions subject to third-party rights. Users must confirm this at their own responsibility before use.

**c.** For Content obtained through API linkage (Application Programming Interface) with external databases and the like, please comply with the usage conditions of the respective providers.

**d.** Even where third parties hold copyrights or other rights, there may be cases where use is permitted without permission under the Copyright Act, such as legally permitted quotation/citation.

**(3) Content to Which These Rules Do Not Apply**

These rules do not apply to symbol marks, logos, and character designs representing organizations or specific projects/programs.

**(4) Governing Law and Jurisdiction**

(Identical in substance to the “Governing Law and Jurisdiction” section above.)

**(5) Disclaimer**

(Identical in substance to the “Disclaimer” section above.)

**(6) Miscellaneous**

**a.** These rules do not restrict uses permitted under the Copyright Act, such as legally permitted quotation/citation.
**b.** These rules were established on April 1, 2020, conform to the Government of Japan Standard Terms of Use (Version 2.0), and may be amended in the future; prior-version conditions continue to apply for existing uses.
**c.** These rules are compatible with CC BY, and Content subject to these rules may also be used under CC BY.

https://creativecommons.org/licenses/by/4.0/legalcode.ja

**Appendix**

**Content Obtained via API Linkage with External Databases, etc.**

The following layers displayed in the Web Mapping System use the Geospatial Information Authority of Japan’s “GSI Tiles.” When using them, please confirm “Regarding Use of GSI Tiles” and comply with its conditions of use.

- Base map
- GSI products (topography and administrative boundaries)
