# Supplementary material for: Spatial analysis of accessibility to healthcare-related facilities in Tokyo Metropolis using geographic information systems
Source: PLoS One. 2026 May 29;21(5):e0350130. doi: 10.1371/journal.pone.0350130 (PMC13221066; doi:10.1371/journal.pone.0350130)
Supplement: S5 File — Digital agency public data license (version 1.0) (Japanese). (PDF) [file pone.0350130.s005.pdf]

公共データ利用規約（第1.0版）

国又は地方公共団体等の公的機関が著作権者である著作物について、広く二次利用を認める形で著作物の利用に対する考えを示すに当たり、できるだけ分かりやすく統一的なものとするため、各府省又は地方公共団体等の公的機関のウェブサイトの利用規約の本文として定めます。

1. 当ウェブサイトのコンテンツの利用について

当ウェブサイトで公開している情報（以下「コンテンツ」といいます。）は、別の利用ルールが適用されるコンテンツを除き、どなたでも以下の1.1から1.7. に定める利用ルール（以下「本利用ルール」といいます。）に従って、複製、公衆送信、翻訳・変形等の翻案等、自由に利用できます（本利用ルールに従って利用できるコンテンツを、以下「本コンテンツ」といいます。）。商用利用も可能です。本コンテンツの利用に当たっては、本利用ルールに同意したものとみなします。
なお、数値データ、簡単な表・グラフ等は著作権による保護の対象ではありませんので、これらについては本利用ルールの適用はなく、自由に利用できます。

1.1. 出典の記載について

1. 本コンテンツを利用する際は出典を記載してください。出典の記載方法は以下の例を参考に、実際の提供元やURL等に置き換えて記載してください。URLリンクが使える場合は（）内のURLは該当する文言からリンクを張る形にすることもできます。また、本コンテンツに係る「公共データ利用規約（第1.0版）に関する重要情報」に出典記載例が示されている場合には、以下の出典記載例の代わりにそちらの記載例を参考にしてください。
   （出典記載例）

   出典：D庁ウェブサイト（当該ページのURL）、PDL1.0（規約原文ページのURL）
   出典：「○○動向調査」（D庁）（当該ページのURL）、PDL1.0（規約原文ページのURL）（○年○月○日に利用）
   なお、上述のように本規約の採用者（国、地方公共団体など）が別に「公共データ利用規約（第1.0版）に関する重要情報」を提示することで変更可能です。

2. 本コンテンツを編集・加工等して利用する場合は、上記出典とは別に、編集・加工等を行ったこと及びその主体を記載してください。また、本コンテンツに係る「公共データ利用規約（第1.0版）に関する重要情報」に該当する記載例が示されている場合には、以下の記載例の代わりにそちらの記載例を参考にしてください。なお、編集・加工した情報を、あたかも国又は府省等（本コンテンツの提供者が地方公共団体等の公的機関の場合はその地方公共団体等の公的機関）が作成した未加工のままであるかのような態様で公

表・利用してはいけません。

（本コンテンツを編集・加工等して利用する場合の記載例）

「○○動向調査」（D庁）（当該ページのURL）を加工して作成
「○○動向調査」（D庁）（当該ページのURL）をもとに○○株式会社作成など

1.2. 第三者の権利を侵害しないようにしてください

1. 本コンテンツの中には、第三者（国以外の者をいいます。本コンテンツの提供者が地方公共団体等の公的機関である場合はその地方公共団体等の公的機関以外の者をいいます。以下同じ。）が著作権その他の権利を有しているものがあります。本コンテンツの内、第三者が著作権を有しているものや、第三者が著作権以外の権利（例：写真における肖像権、パブリシティ権等）を有しているものについては、特に権利処理済であることが明示されているものを除き、利用者の責任で、当該第三者から利用の許諾を得てください。
2. 本コンテンツのうち第三者が権利を有しているものについては、出典の表記等によって第三者が権利を有していることを直接的又は間接的に表示・示唆しているものもありますが、明確に第三者が権利を有している部分の特定・明示等を行っていないものもあります。利用する場合は利用者の責任において確認してください。
   なお、第三者に権利があるコンテンツについて特に注意を喚起したいものがある場合は「公共データ利用規約（第1.0版）に関する重要情報」に記載しています。
3. 外部データベース等とのAPI（Application Programming Interface）連携等により取得しているコンテンツについては、その提供元の利用条件に従ってください。
   なお、外部データベース等とのAPI連携等により取得しているコンテンツについて特に注意を喚起したいものがある場合は「公共データ利用規約（第1.0版）に関する重要情報」に記載しています。
4. 第三者が著作権等を有しているコンテンツであっても、著作権法上認められている引用など、著作権者等の許諾なしに利用できる場合があります。

1.3. 個別法令による利用の制約があるコンテンツについて

本コンテンツの一部には、個別法令により利用に制約がある場合があります。
なお、個別法令による制約について特に注意を喚起したいものがある場合は「公共データ利用規約（第1.0版）に関する重要情報」に記載しています。

1.4. 本利用ルールが適用されないコンテンツについて

以下のコンテンツについては、本利用ルールの適用外です。
なお、別の利用ルールが適用されることが明示されているコンテンツがある場合は「公共データ利用規約（第1.0版）に関する重要情報」に記載しています。

1. 組織や特定の事業を表すシンボルマーク、ロゴ、キャラクターデザイン
2. 具体的かつ合理的な根拠の説明とともに、別の利用ルールが適用されることが明示されているコンテンツ

1.5. 準拠法と合意管轄について

1. 本利用ルールは、日本法に基づいて解釈されます。
2. 本利用ルールによる本コンテンツの利用及び本利用ルールに関する紛争については、当該紛争に係る本コンテンツを公開している組織の所在地を管轄する地方裁判所又は簡易裁判所を、第一審の専属的合意管轄裁判所とします。

1.6. 免責について

1. 国（本コンテンツが国ではなく地方公共団体等の公的機関によって提供されている場合はその地方公共団体等の公的機関）は、利用者が本コンテンツを用いて行う一切の行為（本コンテンツを編集・加工等した情報を利用することを含みます。）について何ら責任を負うものではありません。
2. 本コンテンツは、予告なく変更、移転、削除等が行われることがあります。

1.7. その他

1. 本利用ルールは、著作権法上認められている引用などの利用について、制限するものではありません。
2. 本利用ルールは、令和6年7月5日に定めたものです。本利用ルールは、今後変更される可能性があります。なお、既に以前の政府標準利用規約にしたがってコンテンツを利用している場合は、引き続きその条件が適用されます。
3. 本利用ルールは、クリエイティブ・コモンズ・ライセンスの表示4.0　国際ライセンスに規定される著作権利用許諾条件。以下「CC BY」といいます。）と互換性があります。国（本コンテンツが国ではなく地方公共団体によって提供されている場合はその地方公共団体）は、本利用ルールが適用される本コンテンツについて、利用者がCC BYに従って利用することを許諾します。
4. ウェブサイト全体についてのリンクポリシー、プライバシーポリシー、アクセシビリティや免責事項については、本利用ルールに基づく本コンテンツ利用に係る内容と矛盾しない限り、本利用ルールを採用する国又は地方公共団体等の側で自由に定められます。
5. 本利用ルールは地方公共団体によって提供されるコンテンツの利用ルールとして適用されることもあります。
6. 本利用ルールは、ウェブサイト全体だけでなく、個別のコンテンツに適用されることもあります。
7. 利用規約名の表記において簡略化を図るため「公共データ利用規約（第1.0版）」は「PDL1.0」と表記することがあります（利用者もそのように表記することも可能です。）。なお、PDLは「Public Data License」の頭文字から取ったものです。

デジタル庁

〒102-0094
東京都千代田区紀尾井町1-3
東京ガーデンテラス紀尾井町

電話番号：(03)4477-6775（代表）
法人番号：8000012010038

© Digital Agency,
Government of Japan
