# Supplementary material for: Spatial analysis of accessibility to healthcare-related facilities in Tokyo Metropolis using geographic information systems
Source: PLoS One. 2026 May 29;21(5):e0350130. doi: 10.1371/journal.pone.0350130 (PMC13221066; doi:10.1371/journal.pone.0350130)
Supplement: S6 File — Digital agency public data license (version 1.0) (English). (DOCX) [file pone.0350130.s006.docx]

Digital Agency Public Data License (Version 1.0)

© Digital Agency, Government of Japan

<https://www.digital.go.jp/resources/open_data/public_data_license_v1.0>

(translated by ChatGPT 5.2)

**Public Data License (Version 1.0) (PDL1.0)**

In order to present, in a form that broadly permits secondary use, the policy on the use of works for which the copyright holder is the national government or a local public entity or other public institution, this license is established as the main body of the terms of use for the websites of ministries/agencies or local public entities and other public institutions, in as clear and uniform a manner as possible.

**1. Use of Content on This Website**

The information published on this website (hereinafter referred to as the “Content”), except for Content to which different usage rules apply, may be freely used by anyone—such as by reproduction, public transmission, translation, and adaptation including modification and transformation—in accordance with the usage rules set forth in Sections 1.1 through 1.7 below (hereinafter referred to as these “Usage Rules”). Content that may be used in accordance with these Usage Rules is hereinafter referred to as the “Licensed Content.” Commercial use is also permitted. Users of the Licensed Content are deemed to have agreed to these Usage Rules.

Note that numerical data, simple tables, graphs, and the like are not protected by copyright; therefore, these Usage Rules do not apply to such materials, and they may be used freely.

**1.1 Indication of Source**

When using the Licensed Content, please indicate the source. Please refer to the examples below and replace them with the actual provider, URL, etc. Where URL links can be used, the URL shown in parentheses may alternatively be provided as a hyperlink from the relevant wording. In addition, if “Important Information Regarding the Public Data License (Version 1.0)” for the Licensed Content provides examples of source attribution, please refer to those examples instead of the examples below.

**(Examples of source attribution)**

- Source: Agency D website (URL of the relevant page), PDL1.0 (URL of the page containing the original text of the license)
- Source: “○○ Trend Survey” (Agency D) (URL of the relevant page), PDL1.0 (URL of the page containing the original text of the license) (accessed on: ○○/○○/○○)

As stated above, the adopter of this license (the national government, local public entities, etc.) may modify these examples by separately presenting “Important Information Regarding the Public Data License (Version 1.0).”

If you use the Licensed Content after editing, processing, or otherwise modifying it, please state **that such editing/processing was performed and by whom**, in addition to the source above. If “Important Information Regarding the Public Data License (Version 1.0)” for the Licensed Content provides relevant examples, please refer to those examples instead of the examples below. You must not publish or use edited/processed information in a manner that makes it appear as though it remains unmodified and was created by the national government or ministries/agencies, etc. (or, where the provider of the Licensed Content is a local public entity or other public institution, by that local public entity or other public institution).

**(Examples when editing/processing the Licensed Content)**

- Created by processing “○○ Trend Survey” (Agency D) (URL of the relevant page)
- Created by ○○ Co., Ltd. based on “○○ Trend Survey” (Agency D) (URL of the relevant page), etc.

**1.2 Please Do Not Infringe Third-Party Rights**

Some of the Licensed Content may be subject to copyrights or other rights held by third parties (meaning persons other than the national government; where the provider of the Licensed Content is a local public entity or other public institution, persons other than that local public entity or other public institution; the same applies hereinafter). For Licensed Content in which third parties hold copyrights, or in which third parties hold rights other than copyright (e.g., portrait rights or publicity rights in photographs), unless it is clearly indicated that rights clearance has been completed, users must obtain permission for use from the relevant third parties at their own responsibility.

For Licensed Content in which third parties hold rights, some materials may directly or indirectly indicate or suggest that third parties hold such rights through source indications, etc., while other materials may not clearly identify or specify the portions subject to third-party rights. Users must confirm this at their own responsibility before use.

If there are materials for which particular caution regarding third-party rights is necessary, this is stated in “Important Information Regarding the Public Data License (Version 1.0).”

For Content obtained through linkage with external databases, etc. via an API (Application Programming Interface), please comply with the usage conditions of the respective providers.

If there are materials obtained via API linkage, etc. for which particular caution is necessary, this is stated in “Important Information Regarding the Public Data License (Version 1.0).”

Even where third parties hold copyrights or other rights, there may be cases where use is permitted without permission under the Copyright Act, such as legally permitted quotation/citation.

**1.3 Content Subject to Restrictions Under Individual Laws**

Some of the Licensed Content may be subject to restrictions on use under individual laws.

If there are matters for which particular caution regarding restrictions under individual laws is necessary, this is stated in “Important Information Regarding the Public Data License (Version 1.0).”

**1.4 Content to Which These Usage Rules Do Not Apply**

These Usage Rules do not apply to the following Content.
If it is clearly indicated that different usage rules apply to certain Content, such Content is listed in “Important Information Regarding the Public Data License (Version 1.0).”

- Symbol marks, logos, and character designs representing organizations or specific projects/programs
- Content for which it is clearly stated—together with a specific and reasonable explanation of grounds—that different usage rules apply

**1.5 Governing Law and Jurisdiction**

These Usage Rules shall be interpreted in accordance with the laws of Japan.

With respect to the use of the Licensed Content under these Usage Rules and any disputes relating to these Usage Rules, the district court or summary court having jurisdiction over the location of the organization that publishes the Licensed Content relevant to the dispute shall be the exclusive agreed court of first instance.

**1.6 Disclaimer**

The national government (or, where the Licensed Content is provided not by the national government but by a local public entity or other public institution, that local public entity or other public institution) shall not be liable for any actions taken by users using the Licensed Content (including the use of information created by editing/processing the Licensed Content).

The Licensed Content may be changed, moved, deleted, etc., without prior notice.

**1.7 Miscellaneous**

These Usage Rules do not restrict uses permitted under the Copyright Act, such as legally permitted quotation/citation.

These Usage Rules were established on **July 5, 2024**. These Usage Rules may be amended in the future. If you have already been using the Content in accordance with previous Government Standard Terms of Use, the conditions of those prior terms will continue to apply.

These Usage Rules are compatible with the copyright licensing terms specified in the **Creative Commons Attribution 4.0 International License** (hereinafter “CC BY”). The national government (or, where the Licensed Content is provided not by the national government but by a local public entity, that local public entity) grants users permission to use the Licensed Content to which these Usage Rules apply in accordance with CC BY.

Policies for the website as a whole—such as link policies, privacy policies, accessibility, and disclaimers—may be freely determined by the national government or local public entities, etc. adopting these Usage Rules, insofar as they do not conflict with matters relating to the use of the Licensed Content under these Usage Rules.

These Usage Rules may also apply as rules for using Content provided by local public entities.

These Usage Rules may apply not only to the website as a whole but also to individual Content items.

For simplicity in indicating the name of these terms, “Public Data License (Version 1.0)” may be referred to as “PDL1.0” (users may also do so). “PDL” is derived from the initials of “Public Data License.”
