# Supplementary material for: Spatial analysis of accessibility to healthcare-related facilities in Tokyo Metropolis using geographic information systems
Source: PLoS One. 2026 May 29;21(5):e0350130. doi: 10.1371/journal.pone.0350130 (PMC13221066; doi:10.1371/journal.pone.0350130)
Supplement: S1 File — e-stat terms of use (Japanese). (PDF) [file pone.0350130.s001.pdf]

利用規約

政府統計の総合窓口（e-Stat）利用規約

当サイトで公開している情報（以下「コンテンツ」といいます。）は、どなたでも以下の１）～６）に従って、複製、公衆送信、翻訳・変形等の翻案等、自由に利用できます。商用利用も可能です。

また、数値データ、簡単な表・グラフ等は著作権の対象ではありませんので、これらについては本利用ルールの適用はなく、自由に利用できます。

コンテンツ利用に当たっては、本利用ルールに同意したものとみなします。

１） 出典の記載について

ア コンテンツを利用する際は出典を記載してください。出典の記載方法は以下のとおりです。

（出典記載例）
出典：政府統計の総合窓口(e-Stat)（https://www.e-stat.go.jp/）
出典：「○○調査結果」（A省） など

イ コンテンツを編集・加工等して利用する場合は、上記出典とは別に、編集・加工等を行ったことを記載してください。なお、編集・加工した情報を、あたかも国（又は府省等）が作成したかのような態様で公表・利用してはいけません。

（コンテンツを編集・加工等して利用する場合の記載例）
「○○調査結果」（A省）を加工して作成
「○○調査結果」（A省）を基に○○株式会社作成 など

２） 第三者の権利を侵害しないようにしてください

ア コンテンツの中には、第三者（国以外の者をいいます。以下同じ。）が著作権その他の権利を有している場合があります。第三者が著作権を有しているコンテンツや、第三者が著作権以外の権利（例：写真における肖像権、パブリシティ権等）を有しているコンテンツについては、特に権利処理済であることが明示されているものを除き、利用者の責任で、当該第三者から利用の許諾を得てください。

イ コンテンツのうち第三者が権利を有しているものについては、出典の表記等によって第三者が権利を有していることを直接的又は間接的に表示・示唆しているものもありますが、明確に第三者が権利を有している部分の特定・明示等を行っていないものもあります。利用する場合は利用者の責任において確認してください。

ウ 外部データベース等とのＡＰＩ（Application Programming Interface）連携等により取得しているコンテンツについては、その提供元の利用条件に従ってください。

エ 第三者が著作権等を有しているコンテンツであっても、著作権法上認められている引用など、著作権者等の許諾なしに利用できる場合があります。

３） 本利用ルールが適用されないコンテンツについて

以下のコンテンツについては、本利用ルールの適用外です。

ア 組織や特定の事業を表すシンボルマーク、ロゴ、キャラクターデザイン

イ 具体的かつ合理的な根拠の説明とともに、別の利用ルールの適用を明示しているコンテンツ

(別の利用ルールの適用を明示しているコンテンツは、本利用ルールの別紙に列挙しています。)

４） 準拠法と合意管轄について

ア 本利用ルールは、日本法に基づいて解釈されます。

イ 本利用ルールによるコンテンツの利用及び本利用ルールに関する紛争については、当該紛争に係るコンテンツ又は利用ルールを公開している組織の所在地を管轄する地方裁判所を、第一審の専属的な合意管轄裁判所とします。

５） 免責について

ア 国は、利用者がコンテンツを用いて行う一切の行為（コンテンツを編集・加工等した情報を利用することを含む。）について何ら責任を負うものではありません。

イ コンテンツは、予告なく変更、移転、削除等が行われることがあります。

６） その他

ア 本利用ルールは、著作権法上認められている引用などの利用について、制限するものではありません。

イ 本利用ルールは、平成２８年１月２９日に定めたものです。本利用ルールは、政府標準利用規約（第2.0版）に準拠しています。本利用ルールは、今後変更される可能性があります。既に政府標準利用規約の以前の版にしたがってコンテンツを利用している場合は、引き続きその条件が適用されます。

ウ 本利用ルールは、クリエイティブ・コモンズ・ライセンスの表示 4.0 国際（https://creativecommons.org/licenses/by/4.0/legalcode.jaに規定される著作権利用許諾条件。以下「CC BY」といいます。）と互換性があり、本利用ルールが適用されるコンテンツはCC BYに従うことでも利用することができます。

別紙

別の利用ルールが適用されます。詳細はリンク先のページを御参照ください。

政府統計の総合窓口（e-Stat）のロゴの利用について

公的統計のミクロデータ利用ポータルサイト（miripo）及びミクロデータ利用電子申出窓口（e-Micro）のロゴの利用について

ＡＰＩ機能の利用について

統計地理情報システムの利用について

ミクロデータ利用電子申出窓口（e-Micro）の利用について

調査票情報提供機能の利用について
