# Supplementary material for: Spatial analysis of accessibility to healthcare-related facilities in Tokyo Metropolis using geographic information systems
Source: PLoS One. 2026 May 29;21(5):e0350130. doi: 10.1371/journal.pone.0350130 (PMC13221066; doi:10.1371/journal.pone.0350130)
Supplement: S3 File — National land numerical information download site terms of use (Japanese). (PDF) [file pone.0350130.s003.pdf]

利用規約

国土数値情報（政府標準）　国土数値情報（旧利用約款）　位置参照情報　国土調査　ウェブマッピングシステム

国土数値情報ダウンロードサイトコンテンツ利用規約

国土数値情報ダウンロードサイトコンテンツ利用規約（政府標準利用規約準拠版）

（制定 令和2年4月1日）

1．国土数値情報ダウンロードサイトのコンテンツの利用について

　当国土数値情報ダウンロードサイトで公開している情報（以下「コンテンツ」といいます。）は、どなたでも以下の１）～６）に従って、複製、公衆送信、翻訳・変形等の翻案等、自由に利用できます。商用利用も可能です。また、簡単な表・グラフ等は著作権の対象ではありませんので、これらについては本利用ルールの適用はなく、自由に利用できます。
コンテンツ利用に当たっては、本利用ルールに同意したものとみなします。

１）出典の記載について

　ア　コンテンツを利用する際は出典を記載してください。出典の記載方法は以下のとおりです。

（出典記載例）
出典：国土交通省国土数値情報ダウンロードサイト（当該ページのURL）
出典：「国土数値情報（〇〇データ）」（国土交通省）（当該ページのURL）（〇年〇月〇日取得）

　イ　コンテンツを編集・加工して利用する場合は、上記出典とは別に、編集・加工等を行ったことを記載してください。なお、編集・加工した情報を、あたかも国（又は府省等）が作成したかのような態様で公表・利用してはいけません。

（コンテンツを編集・加工等して利用する場合の記載例）
「国土数値情報（〇〇データ）」（国土交通省）（当該ページのURL）を加工して作成
「国土数値情報（〇〇データ）」（国土交通省）（当該ページのURL）をもとに〇〇株式会社作成

２）第三者の権利を侵害しないようにしてください

　ア　コンテンツの中には、第三者（国以外のものをいいます。以下同じ。）が著作権その他の権利を有している場合があります。第三者が著作権を有しているコンテンツや、第三者が著作権以外の権利（例：写真における肖像権、パブリシティ権等）を有しているコンテンツについては、特に権利処理済であることが明示されているものを除き、利用者の責任で、当該第三者から利用の許諾を得てください。

　イ　コンテンツのうち第三者が権利を有しているものについては、出典の表記等によって第三者が権利を有していることを直接的又は間接的に表示・示唆しているものもありますが、明確に第三者が権利を有している部分の特定・明示等を行っていないものもあります。利用する場合は利用者の責任において確認してください。

　ウ　第三者が著作権等を有しているコンテンツであっても、著作権法上認められている引用など、著作権者等の許諾なしに利用できる場合があります。

３）本利用ルールが適用されないコンテンツについて

以下のコンテンツについては、本利用ルールの適用外です。

　ア　組織や特定の事業を表すシンボルマーク、ロゴ、キャラクターデザイン

　イ　具体的かつ合理的な根拠の説明とともに、別の利用ルールの適用を明示しているコンテンツ

　（別の利用ルールの適用を明示しているコンテンツは、本利用ルールの別紙に列挙しています。）

４）準拠法と合意管轄について

　ア　本利用ルールは、日本法に基づいて解釈されます。

　イ　本利用ルールによるコンテンツの利用及び本利用ルールに関する紛争については、当該紛争に係るコンテンツ又は利用ルールを公開している組織の所在地を管轄する地方裁判所を、第一審の専属的な合意管轄裁判所とします。

５）免責について

　ア　国は、利用者がコンテンツを用いて行う一切の行為（コンテンツを編集・加工等した情報を利用することを含む。）について何ら責任を負うものではありません。

　イ　コンテンツは予告なく変更、移転、削除等が行われることがあります。

６）その他

　ア　本利用ルールは、著作権法上認められている引用などの利用について、制限するものではありません。

　イ　本利用ルールは、令和２年４月１日に定めたものです。本利用ルールは、政府標準利用規約（第2.0版）に準拠しています。本利用ルールは、今後変更される可能性があります。既に政府標準利用規約の以前の版にしたがってコンテンツを利用している場合は、引き続きその条件が適用されます。

　ウ　本利用ルールは、クリエイティブ・コモンズ・ライセンスの表示4.0国際（https://creativecommons.org/licenses/by/4.0/legalcode.jaに規定される著作権利用許諾条件。以下「CCBY」といいます。）と互換性があり、本利用ルールが適用されるコンテンツはCCBYに従うことでも利用することができます。

別のルールを適用するコンテンツ

　コンテンツにより、原典資料の提供者により許諾された利用条件が本利用ルールとは異なる場合があります。このため、国土数値情報個別ページの「利用制限」欄に、本利用ルール以外の利用規約等に準拠する旨が記載されている場合は、その利用規約等に従います。

第三者が著作権その他の権利を有しているコンテンツについて

　国土数値情報個別ページの「このデータの使用許諾条件」欄に以下のように記載されているデータは、原典資料の提供者から、本規約に従って当該データを公開することを許諾されています。

<適用する規約>
国土数値情報ダウンロードサイトコンテンツ利用規約（政府標準規約準拠版）

<利用条件>
適用する利用規約に基づく（オープンデータ）

国土数値情報ダウンロードサービスコンテンツ利用規約

国土数値情報ダウンロードサービスコンテンツ利用規約（旧国土情報利用約款準拠版）

（制定 令和2年4月1日）
（改正 令和2年7月1日）

第1条　著作権等の取り扱いについて

「国土数値情報」は、国土に関する基礎的な空間情報のデータベース集で、基本的に、それぞれに国土交通省不動産・建設経済局以外の原著作権者等がいる著作物、知的財産を原本とし、各権利者の許諾範囲に基づき国土交通省不動産・建設経済局が要素を抽出し位置データを付与した二次著作物です。

よって、それぞれの指標ごとに、原著作者、各種権利者等が存在し、それぞれ許諾の内容が異なるので、それぞれに付置された「データの使用許諾条件」及び日本国内の諸法令を遵守し、原著作権者の権利を侵害しないでください。なお、原著作者、各種権利者等の申し出等により、予告なく公開が停止・終了される場合もあります。

国土交通省不動産・建設経済局は、政府のオープンデータ方針に基づき、商用利用も可能なデータの拡大を目指しており、「国土数値情報」のそれぞれの指標群には、下記の2つのライセンスが表示されています。

（ａ）「商用可」＝出典・加工者等表示のうえ、原著作者等の許諾上、商用目的での利用（複製物の再配布を含む）が可能なもの

（ｂ）「非商用」＝出典・加工者等表示のうえ、原著作者等の許諾上、非商用目的のみでの利用（ただし複製物の再配布を除く）が可能なもの

ただし、行政機関等による公的な目的での使用に際し、利用可能な範囲に不明な点がある場合は、国土情報提供サイト運営事務局にお問い合わせください。

また、「国土数値情報」及びその加工物を利用する全ての人に、データごとのライセンスと、データの不完全性や適用限界、権利の所在、著作権等の取り扱い等を含むこの約款の内容を伝達・継承するようにしてください。

第2条　「国土数値情報」の適用限界等とその継承・伝達について

（１）「国土数値情報」は、それぞれの原典に忠実に、国土地理院発行の２万５千分１地形図またはその電子成果を基図に座標取得をしたもので、それぞれに起因する空間的誤差及び時間的誤差を有します。空間的には原典が住所情報である場合、代表点は街区・大字レベル（概ね数100メートル）の公差を持ち、時間的には１～５年程度のタイムラグを有します。よって、中間加工者や最終利用者に至るまでの全ての利用者は、各指標の適用限界を理解し、目的に合致するかどうかをそれぞれ必ず自己判断してください。また、誤った利用が生じないよう。適用限界を継承・伝達してください。

（２）「国土数値情報」は整備時点に事務局に到達した原典資料をもとに作成しており、原典の不着等により、製品仕様に記載があってもデータの一部の欠落あるいは古いままである場合があります。利用者は原典等と突合確認の上、利用してください。

（３）「国土数値情報」は、国土計画及び土地・不動産等関連業務のために作成されたデータを、副次的に公開しているものですので、ナビゲーションや公共測量等の高度な精度が要求される業務（サービス）、各種証明等には使用することは想定しておりません。また、時間的・空間的精度以外でも、利用者の利用目的に適合しているとは限りませんので、利用者は自らの責任でご自身の利用目的に適合しているかどうかを精査の上ご利用ください。

（４）整備者のみならず、成果物・サービスの最終利用者まで、それぞれの利用前提に記す等により、上記適用限界等を必ず継承・伝達してください。

第3条　出典の記載について

（１）「国土数値情報」を利用する際は出典を記載してください。出典の記載方法は以下のとおりです。

**（出典記載例）**	出典：国土交通省国土数値情報ダウンロードサイト（当該ページのURL）
	出典：国土数値情報（〇〇データ）（国土交通省）（当該ページのURL）（〇年〇月〇日取得）

「国土数値情報」を編集・加工等して利用する場合は、上記出典とは別に、編集・加工等を行ったことを記載してください。なお、編集・加工した情報をあたかも国（又は原典著作者）が作成したかのような態様で公表・利用してはいけません。

**（国土数値情報を編集・加工等して利用する場合の出典記載例）**	「国土数値情報（〇〇データ）（国土交通省）（当該ページのURL）を加工して作成
	「国土数値情報（〇〇データ）（国土交通省）（当該ページのURL）をもとに〇〇株式会社作成

（２）「非商用」の「国土数値情報」であっても、GISによって空間演算した結果（データベースでないもの）等については、出典及び加工者名を明示することによって利用可能です。ただし、利用する国土数値情報がGIS形式やエクセル形式等のデータベースの場合は、原著作者等が有するデータベースの著作権の侵害のおそれがありますので、国土情報ダウンロードサービス事務局にお問い合わせください。

第４条　「国土数値情報」の利用

（１）「国土数値情報」は無償で利用できます。ただし、「国土数値情報」を利用するための通信費等の費用は、利用者の負担となります。

（２）「国土数値情報」の利用に当たっては、本利用規約に同意したものとみなします。

（３）「国土数値情報」について、法令、政令、その他全ての法令、条例等の法規に反する目的・手段・方法で利用することを一切禁じます。また、他人の権利を侵害する目的・手段・方法での利用、公序良俗に反する利用についても一切禁じます。

（４）「国土数値情報」の利用目的及び利用方法については、利用者の判断と責任に委ねられており、国土交通省は一切関与いたしません。

（５）一部の「国土数値情報」については、原典として利用したデータに係る著作権を保護する必要性等から、利用目的若しくは利用方法又はその両方に制約がある場合があります。詳細については個別データの説明に必ず従ってください。

第５条　免責

事由の如何を問わず、「国土数値情報」を利用することにより生じた利用者又は第三者の損害については、利用者がその全ての責任を負うものとし、国土交通省及び原典資料の提供者は一切の責任を負いません。

第６条　その他

（１）「国土数値情報」は、予告なしに内容を変更、削除、又は提供を停止、休止又は中止する場合があります。

（２）本利用規約で示す「国土数値情報」の利用に関しては、日本法及び本利用規約に準拠するものとします。

（３）本利用規約に関連する紛争の管轄裁判所は、東京地方裁判所とします。

（４）データについては、最大限の品質確認を行っておりますが、誤りが含まれる可能性もあります。「国土数値情報」の内容の誤りにお気づきの際は、情報を明記の上、問い合わせ先までご連絡ください。

（５）本利用規約は、予告なしに内容を変更する場合があります。

位置参照情報ダウンロードサービスコンテンツ利用規約

位置参照情報ダウンロードサービスコンテンツ利用規約

（制定 令和2年4月1日）

1．位置参照情報ダウンロードサービスのコンテンツの利用について

　当位置参照情報ダウンロードサービスで公開している情報（以下「コンテンツ」といいます。）は、どなたでも以下の１）～６）に従って、複製、公衆送信、翻訳・変形等の翻案等、自由に利用できます。商用利用も可能です。また、簡単な表・グラフ等は著作権の対象ではありませんので、これらについては本利用ルールの適用はなく、自由に利用できます。　コンテンツ利用に当たっては、本利用ルールに同意したものとみなします。

１）出典の記載について

　ア　コンテンツを利用する際は出典を記載してください。出典の記載方法は以下のとおりです。

（出典記載例）

出典：「位置参照情報ダウンロードサービス」（国土交通省）（当該ページのURL）

　イ　コンテンツを編集・加工して利用する場合は、上記出典とは別に、編集・加工等を行ったことを記載してください。なお、編集・加工した情報を、あたかも国（又は府省等）が作成したかのような態様で公表・利用してはいけません。

**（コンテンツを編集・加工等して利用する場合の記載例）**	「位置参照情報ダウンロードサービス」（国土交通省）（当該ページのURL）を加工して作成
	「位置参照情報ダウンロードサービス」（国土交通省）（当該ページのURL）をもとに〇〇株式会社作成

２）第三者の権利を侵害しないようにしてください

　ア　コンテンツの中には、第三者（国以外のものをいいます。以下同じ。）が著作権その他の権利を有している場合があります。第三者が著作権を有しているコンテンツや、第三者が著作権以外の権利（例：写真における肖像権、パブリシティ権等）を有しているコンテンツについては、特に権利処理済であることが明示されているものを除き、利用者の責任で、当該第三者から利用の許諾を得てください。

　イ　コンテンツのうち第三者が権利を有しているものについては、出典の表記等によって第三者が権利を有していることを直接的又は間接的に表示・示唆しているものもありますが、明確に第三者が権利を有している部分の特定・明示等を行っていないものもあります。利用する場合は利用者の責任において確認してください。

　ウ　第三者が著作権等を有しているコンテンツであっても、著作権法上認められている引用など、著作権者等の許諾なしに利用できる場合があります。

３）本利用ルールが適用されないコンテンツについて

以下のコンテンツについては、本利用ルールの適用外です。

　ア　組織や特定の事業を表すシンボルマーク、ロゴ、キャラクターデザイン

４）準拠法と合意管轄について

　ア　本利用ルールは、日本法に基づいて解釈されます。

　イ　本利用ルールによるコンテンツの利用及び本利用ルールに関する紛争については、当該紛争に係るコンテンツ又は利用ルールを公開している組織の所在地を管轄する地方裁判所を、第一審の専属的な合意管轄裁判所とします。

５）免責について

　ア　国は、利用者がコンテンツを用いて行う一切の行為（コンテンツを編集・加工等した情報を利用することを含む。）について何ら責任を負うものではありません。

　イ　コンテンツは予告なく変更、移転、削除等が行われることがあります。

６）その他

　ア　本利用ルールは、著作権法上認められている引用などの利用について、制限するものではありません。

　イ　本利用ルールは、令和２年４月１日に定めたものです。本利用ルールは、政府標準利用規約（第2.0版）に準拠しています。本利用ルールは、今後変更される可能性があります。既に政府標準利用規約の以前の版にしたがってコンテンツを利用している場合は、引き続きその条件が適用されます。

　ウ　本利用ルールは、クリエイティブ・コモンズ・ライセンスの表示4.0国際（https://creativecommons.org/licenses/by/4.0/legalcode.jaに規定される著作権利用許諾条件。以下「CCBY」といいます。）と互換性があり、本利用ルールが適用されるコンテンツはCCBYに従うことでも利用することができます。

国土調査（土地分類調査・水調査）コンテンツ利用規約

国土調査（土地分類調査・水調査）コンテンツ利用規約

（制定 令和2年4月1日）

1．国土調査（土地分類調査・水調査）のコンテンツの利用について

　当国土調査（土地分類調査・水調査）で公開している情報（以下「コンテンツ」といいます。）は、どなたでも以下の１）～７）に従って、複製、公衆送信、翻訳・変形等の翻案等、自由に利用できます。商用利用も可能です。また、簡単な表・グラフ等は著作権の対象ではありませんので、これらについては本利用ルールの適用はなく、自由に利用できます。

　コンテンツ利用に当たっては、本利用ルールに同意したものとみなします。

１）出典の記載について

　ア　コンテンツを利用する際は出典を記載してください。出典の記載方法は以下のとおりです。

**（出典記載例）**	出典：国土交通省国土調査（土地分類調査・水調査）（当該ページのURL）
	出典：1/50,000土地分類基本調査（地形分類図）「東京西北部」東京都（1998）（国土交通省）（当該ページのURL）

　イ　コンテンツを編集・加工して利用する場合は、上記出典とは別に、編集・加工等を行ったことを記載してください。なお、編集・加工した情報を、あたかも国（又は府省等）が作成したかのような態様で公表・利用してはいけません。

**（コンテンツを編集・加工等して利用する場合の記載例）**	「1/50,000土地分類基本調査（地形分類図）「東京西北部」東京都（1998）」（国土交通省）（当該ページのURL）を加工して作成
	「1/50,000土地分類基本調査（地形分類図）「東京西北部」東京都（1998）」（国土交通省）（当該ページのURL）をもとに〇〇株式会社作成

２）第三者の権利を侵害しないようにしてください

　ア　コンテンツの中には、第三者（国以外のものをいいます。以下同じ。）が著作権その他の権利を有している場合があります。第三者が著作権を有しているコンテンツや、第三者が著作権以外の権利（例：写真における肖像権、パブリシティ権等）を有しているコンテンツについては、特に権利処理済であることが明示されているものを除き、利用者の責任で、当該第三者から利用の許諾を得てください。

　イ　コンテンツのうち第三者が権利を有しているものについては、出典の表記等によって第三者が権利を有していることを直接的又は間接的に表示・示唆しているものもありますが、明確に第三者が権利を有している部分の特定・明示等を行っていないものもあります。利用する場合は利用者の責任において確認してください。

　ウ　第三者が著作権等を有しているコンテンツであっても、著作権法上認められている引用など、著作権者等の許諾なしに利用できる場合があります。

３）個別法令による利用の制約があるコンテンツについて

　ア　一部のコンテンツには、個別法令により利用に制約がある場合があります。特に、以下に記載する法令についてはご注意ください。詳しくはそれぞれのリンク先ページをご参照ください。

　・20万分の1土地分類基本調査及び土地保全基本調査、20万分の1シームレス土地保全図、5万分1都道府県土地分類基本調査、土地履歴調査、災害類型別土地保全調査、主要水系調査、都道府県水調査（利水現況図）、地下水調査（地下水マップ）等の成果については、その背景図として国土地理院の基本測量成果を使用しています。よって、背景図を含む本調査の成果について、測量法に基づく複製・使用を行う場合は、国土地理院の承認を得る必要があります。

測量法に基づく基本測量成果の複製・使用に関する申請について

４）本利用ルールが適用されないコンテンツについて

以下のコンテンツについては、本利用ルールの適用外です。

　ア　組織や特定の事業を表すシンボルマーク、ロゴ、キャラクターデザイン

５）準拠法と合意管轄について

　ア　本利用ルールは、日本法に基づいて解釈されます。

　イ　本利用ルールによるコンテンツの利用及び本利用ルールに関する紛争については、当該紛争に係るコンテンツ又は利用ルールを公開している組織の所在地を管轄する地方裁判所を、第一審の専属的な合意管轄裁判所とします。

６）免責について

　ア　国は、利用者がコンテンツを用いて行う一切の行為（コンテンツを編集・加工等した情報を利用することを含む。）について何ら責任を負うものではありません。

　イ　コンテンツは予告なく変更、移転、削除等が行われることがあります。

７）その他

　ア　本利用ルールは、著作権法上認められている引用などの利用について、制限するものではありません。

　イ　本利用ルールは、令和２年４月１日に定めたものです。本利用ルールは、政府標準利用規約（第2.0版）に準拠しています。本利用ルールは、今後変更される可能性があります。既に政府標準利用規約の以前の版にしたがってコンテンツを利用している場合は、引き続きその条件が適用されます。

　ウ　本利用ルールは、クリエイティブ・コモンズ・ライセンスの表示4.0国際（https://creativecommons.org/licenses/by/4.0/legalcode.jaに規定される著作権利用許諾条件。以下「CCBY」といいます。）と互換性があり、本利用ルールが適用されるコンテンツはCCBYに従うことでも利用することができます。

国土情報ウェブマッピングシステムコンテンツ利用規約

国土情報ウェブマッピングシステムコンテンツ利用規約

（制定 令和2年4月1日）

1．国土情報ウェブマッピングシステムのコンテンツの利用について

　当国土情報ウェブマッピングシステムで公開している情報（以下「コンテンツ」といいます。）は、どなたでも以下の１）～６）に従って、複製、公衆送信、翻訳・変形等の翻案等、自由に利用できます。商用利用も可能です。また、簡単な表・グラフ等は著作権の対象ではありませんので、これらについては本利用ルールの適用はなく、自由に利用できます。

　コンテンツ利用に当たっては、本利用ルールに同意したものとみなします。

１）出典の記載について

　ア　コンテンツを利用する際は出典を記載してください。出典の記載方法は以下のとおりです。

（出典記載例）

出典：国土情報ウェブマッピングシステム（国土交通省）（当該ページのURL）

　イ　コンテンツを編集・加工して利用する場合は、上記出典とは別に、編集・加工等を行ったことを記載してください。なお、編集・加工した情報を、あたかも国（又は府省等）が作成したかのような態様で公表・利用してはいけません。

**（コンテンツを編集・加工等して利用する場合の記載例）**	国土情報ウェブマッピングシステム（国土交通省）（当該ページのURL）を加工して作成
	国土情報ウェブマッピングシステム（国土交通省）（当該ページのURL）をもとに〇〇株式会社作成

２）第三者の権利を侵害しないようにしてください

　ア　コンテンツの中には、第三者（国以外のものをいいます。以下同じ。）が著作権その他の権利を有している場合があります。第三者が著作権を有しているコンテンツや、第三者が著作権以外の権利（例：写真における肖像権、パブリシティ権等）を有しているコンテンツについては、特に権利処理済であることが明示されているものを除き、利用者の責任で、当該第三者から利用の許諾を得てください。

　イ　コンテンツのうち第三者が権利を有しているものについては、出典の表記等によって第三者が権利を有していることを直接的又は間接的に表示・示唆しているものもありますが、明確に第三者が権利を有している部分の特定・明示等を行っていないものもあります。利用する場合は利用者の責任において確認してください。

　ウ　外部データベース等とのAPI（Application Programming Interface）連携等により取得しているコンテンツについては、その提供元の利用条件に従ってください。

　エ　第三者が著作権等を有しているコンテンツであっても、著作権法上認められている引用など、著作権者等の許諾なしに利用できる場合があります。

３）本利用ルールが適用されないコンテンツについて

以下のコンテンツについては、本利用ルールの適用外です。

　ア　組織や特定の事業を表すシンボルマーク、ロゴ、キャラクターデザイン

４）準拠法と合意管轄について

　ア　本利用ルールは、日本法に基づいて解釈されます。

　イ　本利用ルールによるコンテンツの利用及び本利用ルールに関する紛争については、当該紛争に係るコンテンツ又は利用ルールを公開している組織の所在地を管轄する地方裁判所を、第一審の専属的な合意管轄裁判所とします。

５）免責について

　ア　国は、利用者がコンテンツを用いて行う一切の行為（コンテンツを編集・加工等した情報を利用することを含む。）について何ら責任を負うものではありません。

　イ　コンテンツは予告なく変更、移転、削除等が行われることがあります。

６）その他

　ア　本利用ルールは、著作権法上認められている引用などの利用について、制限するものではありません。

　イ　本利用ルールは、令和２年４月１日に定めたものです。本利用ルールは、政府標準利用規約（第2.0版）に準拠しています。本利用ルールは、今後変更される可能性があります。既に政府標準利用規約の以前の版にしたがってコンテンツを利用している場合は、引き続きその条件が適用されます。

　ウ　本利用ルールは、クリエイティブ・コモンズ・ライセンスの表示4.0国際（https://creativecommons.org/licenses/by/4.0/legalcode.jaに規定される著作権利用許諾条件。以下「CCBY」といいます。）と互換性があり、本利用ルールが適用されるコンテンツはCCBYに従うことでも利用することができます。

〇別紙

外部データベース等とのAPI連携等により取得しているコンテンツ

ウェブマッピングシステムに表示される以下のレイヤは国土地理院の「地理院タイル」を使用しています。利用に当たっては「地理院タイルのご利用について」を確認の上、その利用条件に従ってください。

・ベースマップ
・国土地理院の成果（地形・行政界）

All rights reserved. Copyright(c) National Land Information Division, National Spatial Planning and Regional Policy Bureau, MLIT of Japan
