# Supplementary material for: Spatial analysis of accessibility to healthcare-related facilities in Tokyo Metropolis using geographic information systems
Source: PLoS One. 2026 May 29;21(5):e0350130. doi: 10.1371/journal.pone.0350130 (PMC13221066; doi:10.1371/journal.pone.0350130)
Supplement: Attachment — Submitted filename: Review PONE-D-25-58835 (2).pdf [file pone.0350130.s025.pdf]

Report

Manuscript ID: PONE-D-25-58835

Title: Spatial Analysis of Accessibility to Healthcare-Related Facilities in Tokyo Metropolis Using Geographic Information Systems

INTRODUCTION SECTION REVIEW (Lines 37-70)

1. Literature Review Insufficiency

- Only two previous Japanese studies are cited (Wang and Sadahiro; Kajimoto et al.)
- No international literature on healthcare accessibility using GIS
- No discussion of methodological approaches (2SFCA, gravity models, etc.) used elsewhere
- Missing citations on pharmacy accessibility specifically

RECOMMENDATION: Expand literature review to include international GIS-based healthcare accessibility studies and explain why the simpler buffer method was chosen over more sophisticated approaches.

2. Research Questions/Objectives Unclear

- The introduction ends with a general aim "to generate evidence to facilitate..." (line 68) but lacks specific research questions
- No hypotheses stated
- Unclear what specific comparisons or relationships will be tested

3. Conceptual Framework Missing

- No discussion of what "accessibility" means conceptually
- The introduction jumps from problem to methods without theoretical grounding
- Reference [10] (Shen & Tao) on spatial access is cited later but not introduced here

RECOMMENDATION: Add a brief paragraph distinguishing spatial accessibility from other dimensions (affordability, acceptability, availability) and justify the focus on spatial proximity.

4. Policy Context Underdeveloped

- Line 44-45 mentions "critical policy concern" but doesn't elaborate
- No mention of existing Japanese healthcare policies or planning frameworks
- The later reference to secondary healthcare zones (line 75) appears without context

RECOMMENDATION: Briefly introduce Japan's healthcare system structure and relevant policies driving interest in accessibility analysis.

2. MATERIALS AND METHODS (Lines 73-155)

1. STUDY DESIGN SECTION MISSING (Lines 73-74)

The methods jump directly into "Study region and selected healthcare facilities" without describing the overall study design.

RECOMMENDATION: Add a brief study design paragraph covering:

• Study type (cross-sectional spatial analysis)
• Timeframe
• Overall analytical approach
• Software and tools used (ArcGIS Pro is mentioned line 126 but should be introduced earlier)

2. FACILITY SELECTION RATIONALE INCOMPLETE (Lines 88-101)

• Line 88: "The target facilities were of four types..." but no justification WHY these four
• Why exclude dental clinics after extracting them (line 99)?
• No operational definitions: What constitutes a "clinic" vs. "hospital"? Are these based on bed count, services, or legal designation?
• Elderly welfare facilities are defined collectively (lines 89-92) but the heterogeneity of these facilities is problematic for accessibility analysis

RECOMMENDATION:

• Provide clear inclusion/exclusion criteria for each facility type
• Define facilities according to Japanese healthcare classifications
• Justify the exclusion of dental clinics
• Consider analysing elderly welfare facilities separately or excluding them

3. DATA QUALITY AND VALIDATION (Lines 93-101)

• No discussion of data completeness or accuracy
• Geocoding quality not assessed-what was the match rate from CSV Address Matching Service?
• Different data vintages (2020 census, 2022 transportation, 2024 pharmacy) not acknowledged
• No mention of how address errors or missing data were handled

RECOMMENDATION: Add a data quality subsection addressing:

• Geocoding match rates and accuracy
• Handling of unmatched addresses
• Impact of using different data years
• Validation methods (if any)

4. ACCESSIBILITY INDICATORS (Lines 108-127)

• Lines 112-114: The "population coverage rate" is well-defined, but the "facility coverage rate" definition is potentially confusing. It's the proportion of facilities near transportation, not transportation near facilities.

• Line 115: "Walking catchment buffers"-the term "catchment" typically refers to service areas of facilities, but here it seems to mean the area around facilities accessible by walking. Clarify terminology.

• Lines 116-120: The justification for 800m extension is based on low car ownership, but:

- What about bicycle use (very common in Tokyo)?
- What about elderly or mobility-impaired populations who cannot walk 800m?
- Is 800m (about 10 minutes) reasonable for emergency care?

• Line 121: "Equivalent 400 and 800m buffers were created around the public transportation nodes"-equivalent in what sense? The meaning differs: walking to a facility vs. walking to transportation to reach a facility.

• No justification for why the same distances (400m, 800m) are appropriate for both facility access and transportation access. Walking to a bus stop vs. walking to a hospital have different tolerance thresholds.

RECOMMENDATIONS:

• Clarify conceptual distinction between facility catchment areas and transportation service areas

• Consider different buffer distances for transportation nodes (perhaps 250m and 500m as commonly used for bus stops)

• Acknowledge limitations for mobility-impaired populations

• Discuss why cycling is not considered despite being common in Tokyo

• Lines 144-152: The justification for including bus routes but excluding railway lines (lines 148-150) makes sense but is incompletely explained. Bus routes are included because passengers can board anywhere, but:

- Do the bus route data indicate where buses actually stop?
- How were bus routes buffered (centerline? both sides of road?)
- Trains can only board at stations, but railway proximity might still matter for perceived accessibility

• Lines 153-155: "Both the population and facility coverage rates were evaluated comparatively",vague. Comparative how? Between what groups? This should specify:

- Urban vs. rural comparisons
- Comparisons across facility types

- Statistical methods (none mentioned!)

RECOMMENDATIONS:

• Add explicit statistical analysis section

• Describe buffer generation for bus routes more precisely

• Acknowledge and discuss the uniform population distribution assumption

• Specify exactly what comparisons will be made and how

6. SECONDARY HEALTHCARE ZONES CLASSIFICATION (Lines 75-80, Table 1)

• Line 77: "excluding remote islands"-how many facilities/population are excluded? This could be important for understanding coverage.

• Lines 78-80: The classification into "urban" (23 special wards) vs. "rural" (Tama region) is overly simplistic:

- Table 1 shows wide variation within each category
- Southern Kitatama has 11,048.8 people/km² (quite urban)
- Nishitama has only 661.9 people/km² (truly rural)
- This binary classification may obscure important nuances

RECOMMENDATION: Consider a three-tier classification (urban/suburban/rural) based on population density thresholds, or analyze as a continuous variable.

7. MISSING METHODOLOGICAL ELEMENTS

• No sample size justification: While this is a complete enumeration, should discuss statistical power for detecting differences

• No ethical statement: Line "N/A" in ethics section—should briefly justify why ethics approval was not needed (public aggregate data)

• No definition of temporal scope: Data from 2020-2024 but no discussion of appropriate reference period

• No handling of seasonal variations: Bus routes and schedules vary seasonally in Tokyo

• No consideration of operational hours: 24-hour pharmacies vs. those closing at 6 PM have very different practical accessibility

• No pilot testing mentioned: Was the methodology tested on a subset before full implementation?

TABLE 1 (Lines 82-83)

• Column header "Population (n)" suggests a sample, but this is total population, use "Population" or "Total Population"

• Consider adding % of Tokyo total for each zone

• Consider adding age distribution (% elderly) given the focus on healthcare needs
• Area calculation precision seems excessive (63.6 km² is clear enough without decimal)

TABLE 2 (Lines 102-106)
• "Type" column includes both administrative boundaries and facility locations—consider splitting into two tables
• "Creation date" and "Acquisition date" distinction unclear-is "creation" when data were produced and "acquisition" when authors obtained them?
• Some acquisition dates in 2024, census data from 2020-should acknowledge this lag
• Footnote symbols (*, †, ‡) not consistently formatted
• URLs should be provided in full or archived (link rot is common)

FIGURE 1 (Lines 86-87)
Caption: "Created with ArcGIS Pro software", unnecessary detail for figure caption
• No scale bar visible
• Administrative boundaries not clearly distinguished
• Consider adding labels for major geographic features (rivers, mountains) mentioned in text

FIGURE 2 (Line 146)
• "Flowchart" may be misleading-this is more of a conceptual diagram showing spatial overlay operations
• Should specify which analysis (population coverage or facility coverage) is being illustrated
• The areal weighting calculation could be shown more explicitly (formula?)
• Consider adding a small example with actual numbers

2. SOFTWARE AND COMPUTATIONAL DETAILS
ArcGIS Pro version mentioned (line 126) but no details on:
• Specific tools used (Buffer, Spatial Join, etc.)

5. DISCUSSIONS

The Discussions section should be extended and more comparation with similar studies should be mentioned.
