# Supplementary material for: Spatial analysis of accessibility to healthcare-related facilities in Tokyo Metropolis using geographic information systems
Source: PLoS One. 2026 May 29;21(5):e0350130. doi: 10.1371/journal.pone.0350130 (PMC13221066; doi:10.1371/journal.pone.0350130)
Supplement: Attachment — Submitted filename: Review 2_PONE-D-25-58835.pdf [file pone.0350130.s027.pdf]

Review 2

Manuscript ID: PONE-D-25-58835

Title: Spatial Analysis of Accessibility to Healthcare-Related Facilities in Tokyo Metropolis Using Geographic Information Systems

I thank the authors for their thorough response. The revised manuscript is substantially stronger, and the point-by-point reply, with precise page and line references, made re-evaluation straightforward.

The Introduction now includes the international GIS literature (Owen et al.; Luo and Wang; Luo and Qi), an additional Japanese study (Nakamura et al.), a clear conceptual framing of spatial accessibility as one dimension among several, three explicit hypotheses, and the policy context of Japan's secondary medical areas. The rationale for adopting a buffer-based approach is now stated transparently.

The Methods have been restructured into clearly labelled subsections, and the authors have in several instances gone beyond what I requested: dental clinics have been incorporated into the analysis rather than excluded; different buffer distances (400–3200 m for facilities, 250–3000 m for transport) are now used, addressing the differing walking tolerances by destination type; geocoding match rates and coordinate-correction procedures are reported explicitly; and operational definitions based on the Medical Care Act have been added. The renaming of "facility coverage rate" to "facility–transportation proximity rate" resolves the conceptual ambiguity I raised. Most importantly, a formal statistical analysis (Mann–Whitney U and Spearman's correlation) has been added, with results integrated into the Results section. The ethical statement, treatment of seasonal and operating-hour variability, and acknowledgement that no pilot testing was conducted have all been added.

On the urban–rural classification, the authors retained the distinction but complemented it with a continuous-variable analysis using population density and aging rate. This is one of the alternatives I explicitly suggested, and their justification, preserving alignment with the planning framework while capturing intra-metropolitan heterogeneity is convincing.

The revised Tables and Figures address my comments: Table 1 has been relabelled and expanded with % of Tokyo total and aging rate; the original Table 2 has been split appropriately; Figure 1 now includes a scale bar and an inset showing terrain and rivers; and

the new Figure 3 provides both the explicit areal-weighting formula and a worked numerical example.

The Discussion has been meaningfully extended, integrating the supplementary statistical findings and offering a more nuanced reading of bus-stop proximity as a function of stop density rather than functional accessibility per se. The Limitations section now addresses the Euclidean-buffer assumption, mobility constraints, the absence of bicycle modelling, the uniform-population-distribution assumption, and temporal variability of public transport.

The authors have addressed essentially all my comments, and the manuscript is now substantially stronger in conceptual framing, methodological transparency, and statistical rigor.

I have no further substantive concerns and recommend acceptance.
