# Supplementary material for: Spatial analysis of accessibility to healthcare-related facilities in Tokyo Metropolis using geographic information systems
Source: PLoS One. 2026 May 29;21(5):e0350130. doi: 10.1371/journal.pone.0350130 (PMC13221066; doi:10.1371/journal.pone.0350130)
Supplement: Attachment — Submitted filename: Response to Reviewer 2.docx [file pone.0350130.s028.docx]

Response to Reviewer 2

We sincerely thank you for the thorough and constructive review of our manuscript. Your detailed feedback has provided valuable insights that have helped us improve the clarity, rigor, and scholarly contribution of the study. We have addressed each of your comments below, outlining the corresponding revisions and additions made to the manuscript. Our responses are numbered to match your points.

**1. The abstract and introduction motivate the study by referring to rural–urban disparities in healthcare access. However, the empirical analysis focuses exclusively on the Tokyo metropolitan area, a highly urbanised context. The authors should clarify why Tokyo alone is sufficient to address the stated motivation, or revise the framing to better align with an intra-metropolitan accessibility analysis rather than a rural–urban comparison.**

1-1. **The abstract and introduction motivate the study by referring to rural–urban disparities in healthcare access. However, the empirical analysis focuses exclusively on the Tokyo metropolitan area, a highly urbanised context.**

We thank the reviewer for this valuable comment. In the original manuscript, we placed too much emphasis on the urban–rural disparity across Japan as a whole. In the revised manuscript, we have clarified that the comparison was made between secondary medical care areas located within the 23 special wards and those in the Tama region of Tokyo Metropolis. We have also stated that this dichotomous classification was used as an operational approach for a broad comparison, and that the internal heterogeneity within these areas was complemented by analyses using continuous variables. Accordingly, we have made it clear that the present study is an intra-metropolitan spatial accessibility analysis within Tokyo Metropolis.

These revisions have been incorporated into the revised manuscript (page 12, lines 189–200; page 19, lines 281–291).

1-2. **The authors should clarify why Tokyo alone is sufficient to address the stated motivation, or revise the framing to better align with an intra-metropolitan accessibility analysis rather than a rural–urban comparison.**

Please see our response to Comment 1-1 above.

**2. The analysis treats all hospitals as equivalent in terms of service provision. This assumption is problematic, as hospitals differ significantly in size, service scope, and clinical capacity. While line 128 shows that Japan has 3 levels of healthcare regions, the result only shows 1 type of hospital. At a minimum, the authors should more clearly justify this simplification and explicitly limit interpretations of results to spatial proximity rather than to effective healthcare access.**

**2-1. The analysis treats all hospitals as equivalent in terms of service provision. This assumption is problematic, as hospitals differ significantly in size, service scope, and clinical capacity.**

We thank the reviewer for this valuable comment. We acknowledge that treating hospitals as a single category was a simplification, and, as the reviewer correctly pointed out, our explanation in the original manuscript was insufficient. In the revised manuscript, we have explicitly added this point as a limitation. At the same time, we have clarified that this study evaluates not hospital function or service capacity, but rather geographic accessibility to hospitals.

These revisions have been incorporated into the revised manuscript (page 8, lines 138–143; page 35, lines 486–488).

**2-2. While line 128 shows that Japan has 3 levels of healthcare regions, the result only shows 1 type of hospital. At a minimum, the authors should more clearly justify this simplification and explicitly limit interpretations of results to spatial proximity rather than to effective healthcare access.**

Please see our response to Comment 2-1 above.

**3. Accessibility is measured using fixed distance buffers, which may be misleading in a dense and complex urban road network such as Tokyo. Network-based methods are well established in the GIS literature and have been widely applied to healthcare accessibility studies. The authors should justify the choice of Euclidean buffers more carefully, or at least discuss how network-based approaches might alter the results. Furthermore, several publications have implemented the Network Voronoi diagram to identify the catchment area**

**3-1. Accessibility is measured using fixed distance buffers, which may be misleading in a dense and complex urban road network such as Tokyo.**

We thank the reviewer for this valuable comment. In accordance with the reviewer’s suggestion, we have clarified that the present analysis, which used Euclidean buffers, does not fully capture actual travel within Tokyo’s complex road network. We have also added that the results should not be interpreted as behaviorally calibrated walking service areas or as indicators of emergency-care access.

These revisions have been incorporated into the revised manuscript (page 34, lines 472–484).

**3-2. Network-based methods are well established in the GIS literature and have been widely applied to healthcare accessibility studies.**

We thank the reviewer for this valuable comment. We have added Nakamura et al. [12], Owen et al. [13], Luo and Wang [14], and Luo and Qi [15] as relevant references on GIS-based healthcare accessibility, including network-based methods, and have positioned our methodological choice within a broader methodological context.

These revisions have been incorporated into the revised manuscript (page 4, line 75 to page 5, line 7; page 5, lines 84–100).

**3-3. The authors should justify the choice of Euclidean buffers more carefully, or at least discuss how network-based approaches might alter the results.**

We thank the reviewer for this valuable comment. We acknowledge that our previous explanation was insufficient. In the revised manuscript, we have provided a more specific explanation of why we adopted a buffer-based approach. Specifically, drawing on Guagliardo [1] and Boscoe et al. [32], this study was intended as a first-stage descriptive assessment comparing multiple facility types, secondary medical care areas, and distance bands using consistent public data. We therefore adopted this method because of its transparency and reproducibility. In addition, under Future Research Directions, we have added network Voronoi-based catchments and other network-partitioning approaches as methodological directions for future research to better represent more realistic service areas.

These revisions have been incorporated into the revised manuscript (page 5, lines 87–89; page 36, lines 504–514).

**3-4. Furthermore, several publications have implemented the Network Voronoi diagram to identify the catchment area.**

Please see the above response to Comment 3-3.

**4. The study appears to focus largely on calculating population counts within 400 m and 800 m buffers around healthcare facilities and transport nodes. While this approach is simple, the methodological steps would benefit from clearer explanation and visualisation. Given that this is a GIS-focused study, additional schematic figures illustrating the analytical workflow and assumptions would greatly improve clarity.**

**4-1. The study appears to focus largely on calculating population counts within 400 m and 800 m buffers around healthcare facilities and transport nodes. While this approach is simple, the methodological steps would benefit from clearer explanation and visualisation.**

We thank the reviewer for this valuable comment. We have revised the structure of the Methods section and reorganized it into the following subsections: Study design, Data sources, data quality, and geocoding, Accessibility indicators and analytical methods, and Comparative evaluation and statistical analysis. We have also revised the manuscript to explain the facility-centered buffer analysis and the transit-centered buffer analysis separately. In addition, we have prepared a new flowchart and a figure illustrating the area-weighting procedure.

These revisions have been incorporated into the revised manuscript (Figs. 2 and 3).

**4-2. Given that this is a GIS-focused study, additional schematic figures illustrating the analytical workflow and assumptions would greatly improve clarity.**

Please see our response to Comment 4-1 above.

**5. Several figures show expected patterns, such as higher population density and higher facility coverage in central urban areas compared to peripheral areas. While these results are not incorrect, the manuscript would benefit from deeper interpretation. For example, it is unclear whether the observed accessibility levels are adequate relative to population needs, or how they compare across demographic groups.**

**5-1. Several figures show expected patterns, such as higher population density and higher facility coverage in central urban areas compared to peripheral areas. While these results are not incorrect, the manuscript would benefit from deeper interpretation.**

We thank the reviewer for this valuable comment. We have expanded the Discussion to ensure that the results are not interpreted simply in terms of a center–periphery difference. Specifically, in the revised manuscript, we have emphasized that (1) substantial heterogeneity exists even within secondary medical care areas, (2) population density and the aging rate are associated with accessibility indicators, and (3) bus-related proximity and railway-station proximity show different spatial patterns. On this basis, we have stated that the implications of the findings should be interpreted more cautiously.

These revisions have been incorporated into the revised manuscript (page 31, line 405 to page 34, line 468).

**5-2. For example, it is unclear whether the observed accessibility levels are adequate relative to population needs, or how they compare across demographic groups.**

We thank the reviewer for this valuable comment. We have clarified that this study evaluates spatial proximity. We have also stated that differences in facility capacity and service volume are beyond the scope of the present study. In addition, we noted that a challenge for future research will be to make use of network Voronoi-based catchments and other network-partitioning approaches in order to better represent realistic service areas.

These revisions have been incorporated into the revised manuscript (page 35, lines 485–487; page 36, lines 512–514).

**6. The authors acknowledge important limitations, including the use of Euclidean buffers, the lack of facility capacity differentiation, and the exclusion of non-spatial factors such as waiting time or socioeconomic conditions. However, the conclusions still make relatively strong claims about healthcare accessibility. The authors should ensure that the conclusions are more tightly aligned with what the simplified methodology can realistically support.**

**6-1. The authors acknowledge important limitations, including the use of Euclidean buffers, the lack of facility capacity differentiation, and the exclusion of non-spatial factors such as waiting time or socioeconomic conditions.**

We thank the reviewer for this valuable comment. In light of the limitations pointed out by the reviewer, we have reorganized the Limitations section to clarify the following issues: the limitations of Euclidean buffers, the lack of consideration of facility capacity, the lack of consideration of non-spatial factors, the lack of consideration of transport service quality, and the assumption of uniform distribution underlying the area-weighting method.

These revisions have been incorporated into the revised manuscript (page 34, line 471 to page 36, line 501).

**6-2. However, the conclusions still make relatively strong claims about healthcare accessibility.**

We thank the reviewer for this valuable comment. We have moderated the wording of the Conclusion throughout and explicitly stated that the findings obtained in this study reflect spatial proximity only, and do not directly indicate service capacity or facility utilization.

These revisions have been incorporated into the revised manuscript (page 37, lines 517–527).

**6-3. The authors should ensure that the conclusions are more tightly aligned with what the simplified methodology can realistically support.**

We thank the reviewer for this valuable comment. In the Conclusion, we have revised the wording to avoid definitive statements regarding service capacity or actual utilization, and instead to focus on the fact that the present findings represent standardized proxy measures for potential access based on facility-centered population coverage and transport-related facility coverage.

These revisions have been incorporated into the revised manuscript (page 37, lines 517–527).

**7. In the conclusion, the author highlighted an imbalance in accessibility between the railway and the bus, where the bus has more comprehensive coverage. Under the current buffer-based approach, the finding that bus networks provide more comprehensive coverage than rail is effectively predetermined by the spatial density of bus stops. Without accounting for service frequency, capacity, or network connectivity, this conclusion risks overstating the functional accessibility contribution of bus systems.**

**7-1. In the conclusion, the author highlighted an imbalance in accessibility between the railway and the bus, where the bus has more comprehensive coverage.**

We thank the reviewer for this valuable comment. In the original manuscript, we may have overstated the implications of bus coverage. In the revised manuscript, we have clarified that straight-line buffers may lead to overestimation and that the findings do not necessarily mean that buses are superior to railways in terms of functional accessibility.

These revisions have been incorporated into the revised manuscript (page 32, lines 427–436; page 34, lines 473–484).

**7-2. Under the current buffer-based approach, the finding that bus networks provide more comprehensive coverage than rail is effectively predetermined by the spatial density of bus stops.**

Please see our response to Comment 7-1 above.

**7-3. Without accounting for service frequency, capacity, or network connectivity, this conclusion risks overstating the functional accessibility contribution of bus systems.**

Please see our response to Comment 7-1 above.
